# Association between Vitamin D Deficiency and High Serum Levels of Small Dense LDL in Middle-Aged Adults

**DOI:** 10.3390/biomedicines9050464

**Published:** 2021-04-24

**Authors:** Yin-Yi Han, Sandy Huey-Jen Hsu, Ta-Chen Su

**Affiliations:** 1 Department of Anesthesiology, National Taiwan University Hospital, Taipei 100225, Taiwan; yyhan@ntuh.gov.tw; 2 Department of Traumatology, National Taiwan University Hospital, Taipei 100225, Taiwan; 3 Department of Laboratory Medicine, National Taiwan University Hospital, College of Medicine, National Taiwan University, Taipei 100225, Taiwan; sandyhsu@ntuh.gov.tw; 4 Department of Environmental and Occupational Medicine, National Taiwan University Hospital, Taipei 100225, Taiwan; 5 Department of Internal Medicine and Cardiovascular Center, National Taiwan University Hospital, Taipei 100225, Taiwan

**Keywords:** vitamin D, small dense LDL, atherogenic dyslipidemia, cardiovascular diseases, insulin resistance

## Abstract

Recent studies suggested a potential link between vitamin D deficiency and cardiovascular risk factors, including dyslipidemia. This study aimed to investigate the association between serum 25(OH)D levels and atherogenic lipid profiles, specifically, that of small dense low-density lipoprotein-cholesterol (sdLDL-C). From 2009 to 2011, a total of 715 individuals aged 35–65 without evident cardiovascular disease (CVD) were enrolled. Their levels of serum 25(OH)D and lipid profiles were measured. Vitamin D deficiency was found to be more common in females, smokers, alcohol drinkers, individuals at a younger age, and those who do not exercise regularly. The analysis of lipid profiles revealed that high sdLDL-C levels were associated with low serum vitamin D levels and were more common among cigarette smokers; alcohol drinkers; individuals with hypertension; individuals with high BMI; and those with high levels of fasting blood glucose, triglycerides, LDL-C, and VLDL-C. The use of multivariate logistic regression verified a strong negative correlation between low vitamin D status (serum 25(OH)D < 15 ng/mL) and the three identified biomarkers of atherogenic dyslipidemia: high serum levels of sdLDL-C, triglycerides, and VLDL-C. This study provides strong evidence that vitamin D deficiency is associated with atherogenic dyslipidemia, and in particular, high sdLDL-C levels in middle-aged adults without CVD.

## 1. Introduction

Cardiovascular diseases (CVDs) are a leading cause of death globally. The World Health Organization (WHO) estimates that 17.9 million people die each year as a result of CVDs [1]. In Taiwan, the death rate was 12,176 per 100,000 people with CVDs in 2019 [2]. Dyslipidemia is atherogenic and is one of the strongest risk factors for CVDs. Dyslipidemia is characterized by abnormal serum levels of lipids, such as triglycerides and lipoproteins, which are classified based on their density levels: high-density (HDL), ultralow-density (ULDL), very low-density (VLDL), and low-density (LDL) lipoproteins [3]. Many studies have been conducted to determine the association between atherogenic dyslipidemia and CVD. Toth et al. [4] reported that the rate of new diagnoses of heart failure is 24% higher in the cohort with triglycerides > 200 mg/dL. In a study of 1324 patients with ambulatory peritoneal dialysis, Xie et al. [5] found that the hazard ratio was 2.08 for all-cause mortality and 1.92 for CVD mortality in patients with VLDL-C levels > 37.5 mg/dL compared to those with VLDL-C levels < 24.4 mg/dL. In an 11-year study of a cohort in Taiwan, a very high risk of coronary heart disease was observed in people with low HDL-C, high body mass index (BMI), or metabolic syndrome [6].

Many factors may affect serum lipid levels; one in particular is that of gender. In a five-year follow-up study of 3602 people in Taiwan, we found that men are more likely than women to have serum levels with HDL-cholesterol (HDL-C) < 35 mg/dL but are less likely to have LDL-cholesterol (LDL-C) levels ≥ 160 mg/dL [7]. Furthermore, we found that postmenopausal women have increased serum levels of triglycerides and decreased serum levels of HDL [8]. It has also been shown that dyslipidemia is more common in individuals with vitamin D deficiency [9].

In addition to enhancing calcium absorption via the intestines and maintaining skeletal health [10], vitamin D affects the functions of many non-skeletal tissue cells including cardiomyocytes [11], vascular wall cells [12,13], and immune cells [14] via paracrine and autocrine mechanisms and therefore plays an important role in cardiovascular health. Vitamin D is mainly produced by the skin upon exposure to sunlight, and its presence in humans is determined by measuring the serum levels of 25(OH)D. Vitamin D deficiency is very common because many people have insufficient exposure to sunlight. The prevalence of vitamin D deficiency is estimated to be between 18% and 84% in various regions of the world and among ethnic populations with different dietary habits [15,16].

Many studies have shown an association between vitamin D deficiency and dyslipidemia [17]. A very significant result of the effect of vitamin D on lipid profile was reported by Ponda et al. [18]. In their study, a group of 108,711 subjects with different serum 25(OH)D and lipid measurements taken from 4 to 26 weeks apart were selected from among four million laboratory test results. Investigations of these subjects revealed that higher serum levels of 25(OH)D correlated with lower levels of total cholesterol, LDL-C, and triglycerides and higher levels of HDL-C in both men and women [18].

Low HDL-C levels; a total cholesterol to HDL-C (T-CHO/HDL-C) ratio greater than five; and high levels of LDL-C, triglycerides, non-HDL-C (total cholesterol minus HDL-C), and apolipoprotein-B (ApoB) are all conventionally considered biomarkers of atherosclerosis [19]. Although high LDL-C levels are a major risk factor for CVDs and the primary target of lipid-lowering therapy, many individuals with normal LDL-C levels still develop CVDs [20]. LDL particles are heterogeneous and are further classified as large buoyant (lb), intermediate, and sdLDL [21]. In hypertriglyceridemia (>200 mg/dL), apoC3-containing lipoproteins are secreted by the liver and may be converted to sdLDL, which remain in the blood for a longer period of time [21]. The circulating sdLDL readily undergoes modifications such as desialylation, glycation, and oxidation. Since modified sdLDL is a potent inducer of inflammation, it is highly atherogenic [22,23] and is associated with a number of diseases, such as metabolic disorders [24,25], obesity [26,27], type 2 diabetes [28,29], and coronary heart disease [30]. It has been previously shown that the rate of coronary heart disease is significantly increased (hazard ratio 2.76–9.98; average, 5.13) in individuals with sdLDL-C levels > 35 mg/dL [31]. A recent study revealed that the elevated levels of sdLDL-C more accurately predict the risk of CVDs than any other atherogenic biomarkers [32].

We have recently investigated the association between the serum levels of sdLDL-C and atherosclerosis risk markers such as inflammation, thrombosis, hematological disorders, and prediabetes; we found that sdLDL-C is a powerful CVD risk biomarker [33]. As vitamin D has been shown to affect lipid profiles, we hypothesized that it may affect serum sdLDL-C levels and then performed a clinical study to test this hypothesis.

## 2. Materials and Methods

### 2.1. Study Design and Population

A total of 715 adults (527 males and 188 females), aged 35–65, were voluntarily enrolled as study subjects at the National Taiwan University Hospital (NTUH) during 2009–2011. Individuals with physician-diagnosed coronary heart disease, heart failure, or cerebrovascular disease were excluded. This study was approved by the Institutional Review Board of the National Taiwan University Hospital. Informed consent was obtained from each subject before enrollment.

### 2.2. CVD Risk Factors and Anthropometric Assessments

Blood pressure (BP) measurements were performed using a mercury sphygmomanometer in a standardized fashion. Two measurements were taken after 5 min of rest in a sitting position. Subjects with systolic blood pressure ≥ 140 mmHg or diastolic BP ≥ 90 mmHg were considered hypertensive [34]. Lifestyle information such as alcohol consumption, smoking, and exercise was collected from self-reported questionnaires. Smokers were those who smoked tobacco regularly, and those that were alcohol drinkers had two or more alcoholic beverages per week. The demographic and anthropometric data of patients were obtained from the medical record archive of NTUH. The body mass index (BMI) of participants was calculated by dividing body weight in kilograms by height in meters squared. Waist circumference was measured in centimeters. Obesity was defined as BMI ≥ 27 kg/m^2^ according to the guidelines of the Health Promotion Administration (MOHW) of Taiwan [35].

### 2.3. Determination of Serum Levels of Vitamin D, Lipid Profile, and Glucose Tolerance

The serum 25(OH)D levels were measured to determine the vitamin D status of subjects. The measurement was performed by liquid chromatography-tandem mass spectrometry (LC-MS/MS), as previously described [36]. The serum levels of T-CHO, triglycerides, HDL-C, and LDL-C were measured using a homogeneous enzymatic method with a commercial kit (Denka Seiken) and were conducted on a Toshiba FR-200 automatic chemistry analyzer (Toshiba, Tokyo, Japan) [37]. The VLDL-C levels were determined by agarose gel electrophoresis, as previously described [37,38]. The plasma sdLDL-C levels were determined using the method of sd-LDL-EX “SEIKEN” on a Toshiba FR-200 automatic chemistry analyzer (Toshiba, Tokyo, Japan), as previously described [39]. The fasting blood glucose levels were measured after at least 8 h of fasting, and plasma glucose concentrations were determined by using a hexokinase assay kit and analyzed on a Toshiba FR-120 automatic chemistry analyzer (Toshiba). The subjects without evident diabetes mellitus received an oral glucose tolerance test (OGTT). After overnight fasting, each subject was asked to drink 300 mL of glucose water containing 75 g of glucose. Venous blood samples were obtained before (OGTT0) and after every 30 min for 120 min (OGTT 120) after the oral intake of glucose. Blood glucose levels at OGTT 120 < 140 mg/dL (7.78 mmol/L), 140–199 mg/dL (7.78–11.06 mmol/L), and >200 mg/dL (11.11 mmol/L) were classified as normal, impaired glucose tolerance (IGT), and diabetic, respectively [33].

### 2.4. Statistical Analysis

Continuous variables such as serum levels of the total cholesterol, triglycerides, glucose, HDL-C, LDL-C, and VLDL-cholesterol (VLDL-C) are expressed as mean ± standard deviation, and categorical data such as the number of smokers and the number of alcohol drinkers are expressed as percentages. The Cochran–Armitage trend test was performed to determine the associations between sdLDL-C and various biomarkers, such as BMI; blood pressure; and the levels of vitamin D, glucose, total cholesterol, triglycerides, HDL-C, LDL-C, and VLDL-C. The significant variables were further analyzed by multivariate logistic regression to determine the correlation (odds ratios) between vitamin D deficiency and atherogenic dyslipidemia variables. This includes triglycerides ≥ 200 mg/dL, sdLDL-C ≥ 40 mg/dL, non-HDL-C ≥ 130 mg/dL, total cholesterol (T-CHO) to HDL-C ratio > 5, and VLDL-C ≥ 30 mg/dL. All analyses were performed using SAS 9.1 (SAS Institute Inc., Cary, NC). A *p* value < 0.05 was considered significant.

## 3. Results

### 3.1. Association between Serum Vitamin D Status and CVD Risk Factors

By comparing the two groups of individuals with serum 25(OH)D levels < 15 ng/mL and ≥15 ng/mL, we found that smoking and alcohol consumption had no significant correlation with serum 25(OH)D levels. Males appeared to have higher vitamin D levels than women, since the male to female ratio in the group with 25(OH)D levels ≥ 15 ng/mL was higher than the group with 25(OH)D levels < 15 ng/mL (75.6% vs. 68%). A larger percentage of individuals (55.5% vs. 37%) who regularly exercised were in the group with 25(OH)D levels ≥ 15 ng/mL. Individuals with lower serum 25(OH)D levels were found to have a higher systolic blood pressure and, hence, a higher risk of hypertension (37% vs. 27.2%). The younger participants were found to have lower serum 25(OH)D levels. There were no significant differences in anthropometric assessments, fasting blood glucose and insulin levels, homeostatic model assessment of insulin resistance (HOMA-IR), HbA1c, OGTT_area under curve (AUC), and OGTT diabetes found between the two groups of subjects. However, a trend of a higher prevalence of obesity and dysglycemia in the group with 25(OH)D levels < 15 ng/mL was noted (Table 1).

### 3.2. Association between Serum sdLDL-C Levels and Dyslipidemia Risk Factors

An analysis of the relationship between serum sdLDL-C levels and vitamin D status was performed by stratifying the data into four sdLDL-C quartiles with a similar number of subjects in each: quartile 1, <22.9 mg/dL; quartile 2, 22.9–33.6 mg/dL; quartile 3, 33.7–47.7 mg/dL; and quartile 4, >47.7 mg/dL. When a comparison was made between quartile 1 and quartile 4, vitamin D was found to have a positive impact on sdLDL-C levels as 79% of patients in quartile 1 had 25(OH)D levels ≥ 15 ng/mL while only 69% in quartile 4 had levels ≥ 15 ng/mL. Both smoking and alcohol consumption were found to have a negative impact on sdLDL-C levels as the percentage of smokers or alcohol drinkers was higher in quartile 4 than in quartile 1. Regular exercise was found to be beneficial as there were more individuals (59%) who exercised regularly in quartile 1 than in quartile 4 (40%). As expected, high sdLDL-C levels were correlated with hypertension. Aging was found to affect sdLDL-C levels in older participants that have higher sdLDL-C levels. Individuals with high BMI were found to have higher sdLDL-C levels. Blood pressure (both SBP and DBP); fasting blood glucose levels; and the levels of T-CHO, triglycerides, LDL-C, and VLDL-C were higher in individuals with higher levels of sdLDL-C. However, HDL-C levels were observed to be inversely proportional to sdLDL-C levels (Table 2).

### 3.3. Association between Severe Vitamin D Deficiency and Increased Risk for Dyslipidemia

The relationship between serum vitamin D levels and lipid profile was investigated by the method of multivariate logistic regression with four different models that were adjusted for various confounding factors. The odds ratios of individuals with serum 25(OH)D levels < 15 ng/mL were compared to those of the individuals with serum 25(OH)D levels ≥ 15 ng/mL. The results of the analyses with models 2, 3, and 4 showed that serum 25(OH)D levels < 15 ng/mL were a significant risk factor for dyslipidemia with sdLDL-C ≥ 40 mg/dL, triglyceride ≥ 200 mg/dL, and VLDL-C ≥ 30 mg/dL, while no significant association was found between vitamin D deficiency and T-CHO/HDL-C ratio > 5, and non-HDL-C > 130 mg/dL (Table 3). In model 1, T-CHO/HDL-C ratio > 5 had a significant correlation with vitamin D deficiency.

## 4. Discussion

In this study of a Taiwanese population, we found that females, smokers, alcohol drinkers, and individuals who do not exercise regularly had higher risk for vitamin D deficiency (Table 1). In addition, vitamin D deficiency was correlated with a higher risk for dyslipidemia including high levels of sdLDL-C (≥40 mg/dL), high levels of triglycerides (≥200 mg/dL), and high levels of VLDL-C (≥30 mg/dL) determined by multivariate regression analyses (Table 3). The high levels of sdLDL-C, triglycerides, and VLDL-C have been shown to be strongly associated with CVDs in previous literature [4,5,31]. Our data also revealed a higher risk for hypertension and dysglycemia in individuals with vitamin D deficiency. Many clinical studies have shown that individuals with vitamin D deficiency have a higher risk of hypertension [40], which is more common in the elderly. Vitamin D deficiency has also been associated with an increased risk of preeclampsia [41], which is characterized by hypertension and endothelial dysfunction in pregnant women. The results of another meta-analysis suggest that the risk of hypertension is lowered by 12% for every 10 ng/mL increment in the circulating 25(OH)D levels [42].

Another significant finding in this study is that the serum sdLDL-C levels were higher in individuals with hypertension; with high BMI; with low serum vitamin D levels; and with high levels of fasting blood glucose, triglycerides, LDL-C, and VLDL-C. Individuals who smoked or consumed alcohol regularly also had higher levels of sdLDL-C, while those who regularly exercised and those with high HDL-C levels had lower sdLDL-C levels (Table 2). In a large prospective study that was conducted on 11,419 individuals, sdLDL-C predicted the risk of coronary heart disease even in those patients considered to be of low CVD risk based on their LDL-C levels [43]. Another study demonstrated that sdLDL-C is a superior marker compared to total LDL-C for the assessment of coronary heart disease [32].

The correlation between vitamin D deficiency and high levels of sdLDL revealed in this study suggests that vitamin D has the potential to decrease sdLDL levels. However, the specific mechanisms of such an action on sdLDL are unknown. Many studies have been performed to determine the mechanisms by which vitamin D affects lipid profiles. One study investigated the effect of vitamin D on gene expression. The active form of vitamin D, 1,25(OH)_2_D3, is the ligand of vitamin D receptor (VDR), which forms a heterodimer with the retinoic acid receptor (RXR). The resulting complex is a nuclear receptor, and it binds to the vitamin D responsive element (VDRE), which is present in many genes, to regulate the expression of genes. By using the Affymetrix Hu133A oligonucleotide microarray, Wang et al. found a total of 913 vitamin D target genes in Hu133A cells (derived from human small lung cell carcinoma) with 734 genes induced and 179 genes repressed by 1,25(OH)_2_D3 treatment [44]. It is conceivable that the expression of many genes related to lipid synthesis and metabolism is regulated by vitamin D.

Chang and Kim [45] showed that the treatment of adipocyte-like 3T3-L1 cells with 100 nmol/L of 1,25(OH)_2_D for 24 h resulted in decreased intracellular fat accumulation and increased lipolysis. A further investigation revealed that such a treatment decreased the expression of adipogenic genes including *αP2, CEBPα, FAS, PPARγ*, and *SCD-1* but increased the expression of lipoprotein lipase (LPL), hormone-sensitive lipase (HSL), and genes involved in β-oxidation of fatty acids [45]. Vitamin D treatment also increased the expression of SIRT1, which increases the mobilization of free fatty acids from adipose tissues [46]. By regulating the expression of genes involved in lipid metabolism, vitamin D increases HDL-C levels, reduces fatty acid synthesis, and enhances β-oxidation of fatty acid, thus lowering triglyceride levels [47]. Vitamin D also modulates serum triglyceride levels through non-genetic mechanisms. A major function of vitamin D is the enhancement of calcium absorption from the intestine. As a result of increased calcium levels, hepatic triglyceride formation and secretion are reduced [48].

Vitamin D deficiency is also associated with insulin resistance, which is a characteristic feature of metabolic syndrome (MetS). Our observation of obesity and dysglycemia being more prevalent in individuals with vitamin D deficiency (Table 1) indicates an association between vitamin D deficiency and MetS. This association has also been demonstrated in other studies. In a study of Korean postmenopausal women, low serum vitamin D3 levels were correlated with features of MetS such as hypertriglycerides and hypertension [49]. With a cohort of 4164 Australians, Gagnon et al. found that the lower 25(OH)D levels were associated with an increased risk of MetS [50]. Using L6 rat myoblasts, Tamilselvan et al. showed that treatment of the cells with vitamin D3 resulted in the increased production of VDR, insulin receptors (IRs), GLUT1, and GLUT4, all of which are related to insulin resistance [51]. Vitamin D also affects insulin resistance by regulating the production of the parathyroid hormone, which in turn increases intracellular Ca++ concentration while leading to decreased GLUT4 activity and the reduced uptake of glucose [52]. The regulation of adiponectin production is another important function of vitamin D. Adiponectin is an adipokine produced by the adipose tissue and has been shown to enhance fatty acid oxidization in muscle cells and fat cells, glucose uptake, and metabolism by muscle cells [53]. Its levels are inversely correlated with obesity and insulin resistance and positively correlated with vitamin D levels [54].

Since vitamin D3 deficiency is common in patients with CVDs or who are at risk for CVDs, we recommend performing proactive prevention by methods such as adequate sunlight exposure, vitamin D3 supplementation, and the intake of vitamin D-rich foods such as fish, eggs, and vitamin D-fortified milk and juice. We also recommend individuals with the following factors be tested for sdLDL-C levels: angina, personal or family history of myocardial infraction, hypertension, high levels of triglycerides and glucose, low HDL-C levels, and obesity.

A weakness of this study is that the effects of diet on lipid profiles and serum vitamin D levels were not investigated. However, such effects have been determined by several previous studies. A study of Australian diets revealed that meat, chicken, fish, egg, and dairy products contribute approximately 4 μg of vitamin D per day in adults >18 years old [55]. In the EPIC-Oxford study of 1388 meat-eaters, 210 fish-eaters, 420 vegetarians, and 89 vegans aged 20–76 years, serum 25(OH)D levels were found to be lower in vegetarians and vegans than in meat- and fish-eaters [56]. In a study of 3257 men and 3551 women in Taiwan, vegan diets were associated with lower HDL-C concentrations in both men and women, and the ovo-lacto vegetarian diet was associated with lower serum LDL-C levels in men [57]. The results of another study of Taiwanese vegetarians and omnivores showed that a vegetarian diet is associated with the lower risk of diabetes and impaired glucose metabolism in both men and women [58]. We previously found that, while the levels of fasting blood glucose, LDL-C, and vitamin B12 are significantly lower in vegetarians, homocysteine and soluble vascular cell adhesion molecule-1 are higher in vegetarians than in omnivores [59]. A second weakness of this study is that we only compared the lipid profile of individuals with severe vitamin D deficiency (serum 25(OH)D levels < 15 ng/mL) to those of individuals with serum 25(O)D3 levels > 15 ng/mL. It has been suggested that serum 25(OH)D levels < 20 ng/mL, 20–30 ng/mL, and >30 ng/mL are classified as deficient, insufficient, and sufficient, respectively [60]. In future studies, we will investigate the correlation between the lipid profile and the three different serum vitamin D levels with a larger cohort of subjects.

In conclusion, our data provide strong evidence that vitamin D deficiency is associated with atherogenic dyslipidemia, particularly high sdLDL-C level, which is a significant CVD risk biomarker.

## Figures and Tables

**Table 1:** The relationship between serum 25(OH)D levels and CVD risk factors.

Variable	25(OH)D		*p* Value
	<15 ng/mL (*n* = 182)	≥15 ng/mL (*n* = 533)	
Male	124 (68%)	403 (75.6%)	0.05
Obesity	51 (28%)	118 (22.1%)	0.06
Smoker	21 (12%)	89 (16.7%)	0.10
Alcohol drinker	34 (19%)	69 (13.0%)	0.06
Exercise	68 (37%)	296 (55.5%)	<0.0001
Hypertension	68 (37%)	145 (27.2%)	0.01
OGTT diabetes mellitus	23 (13%)	63 (11.8%)	0.77
Age	46 ± 10	49 ± 10	0.0006
BMI (kg/m^2^)	25 ± 4	25 ± 3	0.60
Waist (cm)	86 ± 10	85 ± 9	0.39
SBP (mmHg)	127 ± 16	123 ± 13	0.01
DBP (mmHg)	78 ± 11	76 ± 9	0.06
Glucose ^a^ (mg/dL)	98 ± 25	95 ± 17	0.18
Insulin (μIU/mL)	8.2 ± 6.1	7.7 ± 7.7	0.36
HOMA-IR	2.1 ± 1.8	1.9 ± 2.5	0.37
HbA1c (%)	5.8 ± 1.0	5.7 ± 0.7	0.08
OGTT_AUC	17,422 ± 6321	16,689 ± 5082	0.16

Categorical data are expressed as a number (percentage), and the continuous variables are expressed as mean ± standard deviation. Abbreviation: CVD, cardiovascular disease; BMI, body mass index; SBP, systolic blood pressure; DBP, diastolic blood pressure; HOMA-IR, homeostatic model assessment of insulin resistance; OGTT, oral glucose tolerance test; AUC, area under the curve. ^a^ Fasting glucose levels.

**Table 2:** The relationship between sdLDL-C levels and CVD risk factors.

Variable	Serum sdLDL-C Level (mg/dL)				*p*1 Value	*p*2 Value
	<22.9 (*n* = 178)	22.9–33.6 (*n* = 180)	33.7–47.7 (*n* = 179)	>47.7 (*n* = 178)		
Serum 25(OH)D ≥ 15 ng/mL	140 (79%)	140 (78%)	130 (73%)	123 (69%)	0.02	0.04
Smoker	22 (12%)	26 (14%)	27 (15%)	35 (20%)	0.06	0.01
Alcohol drinker	18 (10%)	26 (14%)	23 (13%)	36 (20%)		0.008
Exercise	105 (59%)	102 (57%)	85 (48%)	72 (40%)	0.0004	0.0005
Hypertension	42 (24%)	44 (24%)	62 (35%)	65 (36%)	0.002	0.06
Age	46 ± 9	48 ± 10	48 ± 10	50 ± 10		0.0001
BMI (kg/m^2^)	24 ± 3	25 ± 4	25 ± 3	26 ± 4		<0.0001
SBP (mmHg)	120 ± 14	120 ± 12	130 ± 15	130 ± 13		<0.0001
DBP (mmHg)	72 ± 10	75 ± 9	79 ± 11	79 ± 9		<0.0001
Glucose ^a^ (mg/dL)	90 ± 11	96 ± 24	97 ± 19	100 ± 21		<0.0001
T-CHO (mg/dL)	190 ± 29	210 ± 27	220 ± 32	250 ± 49		<0.0001
TG (mg/dL)	130 ± 230	160 ± 190	230 ± 200	310 ± 430		<0.0001
HDL-C (mg/dL)	52 ± 13	49 ± 13	44 ± 11	46 ± 9		<0.0001
LDL-C (mg/dL)	100 ± 27	120 ± 30	120 ± 35	140 ± 45		<0.0001
VLDL-C (mg/dL)	20 ± 14	23 ± 16	32 ± 17	34 ± 17		<0.0001

Categorical data are expressed as a number (percentage), and the continuous variables are expressed as mean ± standard deviation. *p*1 value: trend test. *p*2 value: sdLDL-C quartile 4 vs. quartile 1. Abbreviations: CVD, cardiovascular disease; BMI, body mass index; SBP, systolic blood pressure; DBP, diastolic blood pressure. ^a^ Fasting glucose levels.

**Table 3:** Determination of the correlation between vitamin D deficiency and atherogenic dyslipidemia variables by multivariate regression analysis ^a^.

**Outcome**	***n***	**Model 1 ^c^**			**Model 2 ^d^**		
		**OR**	**95% CI**	***p* Value**	**OR**	**95% CI**	***p* Value**
sdLDL-C ≥ 40 mg/dL	715	1.74	1.22–2.47	0.002	1.57	1.09–2.27	0.02
TG ≥ 200 mg/dL	715	1.73	1.21–2.48	0.003	1.68	1.14–2.47	0.01
VLDL-C ≥ 30 mg/dL	518 ^b^	1.81	1.21–2.70	0.004	1.82	1.19–2.77	0.01
T-CHO/HDL-C > 5	715	1.49	1.05–2.13	0.03	1.42	0.98–2.07	0.07
non-HDL-C > 130 mg/dL	715	1.09	0.69–1.71	0.72	0.95	0.59–1.52	0.81
**Outcome**	***n***	**Model 3 ^e^**			**Model 4 ^f^**		
		**OR**	**95% CI**	***p* Value**	**OR**	**95% CI**	***p* Value**
sdLDL-C ≥ 40 mg/dL	711	1.47	1.01–2.14	0.04	1.51	1.04–2.19	0.03
TG ≥ 200 mg/dL	711	1.57	1.06–2.34	0.03	1.62	1.09–2.40	0.02
VLDL-C ≥ 30 mg/dL	514 ^b^	1.74	1.14–2.68	0.01	1.78	1.16–2.73	0.01
T-CHO/HDL-C > 5	711	1.30	0.89–1.92	0.18	1.33	0.90–1.95	0.15
non-HDL-C > 130 mg/dL	711	0.85	0.53–1.39	0.52	0.93	0.58–1.49	0.75

^a^ Dummy variable, 0: ≥15, 1: <15. ^b^ Fewer subjects chose not to have VLDL-C levels determined because of the cost. ^c^ Model 1: adjusted for age and sex. ^d^ Model 2: adjusted for age, sex, BMI, cigarette smoking (yes vs. no), alcohol drinking (yes vs. no), and exercise (yes vs. no). ^e^ Model 3: adjusted for age, sex, BMI, cigarette smoking (yes vs. no), alcohol drinking (yes vs. no), exercise (yes vs. no), SBP, and fasting glucose level. ^f^ Model 4: adjusted for age, sex, BMI, cigarette smoking (yes vs. no), alcohol drinking (yes vs. no), exercise (yes vs. no), hypertension, and fasting glucose level. OR, odds ratio; CI, confidence interval.

## Data Availability

All of the relevant data are presented in this article. The raw data are available upon request.
